# Effect of silkworm peptide on inducting M1 type polarization and Th1 activation via TLR2‐induced MyD88‐dependent pathway

**DOI:** 10.1002/fsn3.954

**Published:** 2019-03-12

**Authors:** Guanglai Zhu, Zhongzheng Gui

**Affiliations:** ^1^ School of Biotechnology Jiangsu University of Science and Technology Zhenjiang China; ^2^ Department of Aquatic Science and Technology Jiangsu Animal Husbandry and Veterinary College Taizhou China; ^3^ Sericultural Research Institute Chinese Academy of Agricultural Sciences Zhenjiang China

**Keywords:** CD4^+^ cells, Lewis lung cancer, M1 type polarization, silkworm peptide, Th1 activation, TLR2‐induced MyD88‐dependent pathway

## Abstract

The aim of this study was to explore immune activity and molecular mechanism of silkworm peptide. The cell subsets induced by silkworm peptides were detected by flow cytometry. The IFN‐γ and IL‐4 levels in CD4^+^ cells were measured by ELISA. TLR2 mRNA expression in mouse CD4^+^ T cells was detected by qRT‐PCR. Western blot was used to detect the protein expression levels of MyD88 and p‐IκB. The growth rate of Lewis lung cancer xenografts in mice of the medium‐dose group was significantly reduced, and the tumor volume was significantly smaller than that of the control group on the 14th day. The relative vitality values of spleen lymphocytes in the medium‐dose and high‐dose groups were higher than the control group. The IFN‐γ levels in the medium‐dose and high‐dose groups were significantly higher than the control group. The levels of IL‐4 were no significant change among different groups. Compared with the control group, different doses of silkworm peptide groups could increase the levels of NO, IL‐6, IL‐12, and IL‐1β. Compared with the control group, the protein expression levels of MyD88 and p‐IκB in 10 μg/ml group and 20 μg/ml groups were significantly increased compared with the control group. Silkworm peptide could induce Th1 activation and M1 type polarization, which was dose‐dependent and was relative to the effect of silkworm peptide on inhibiting tumor growth. Silkworm peptide could directly induce M1 type polarization and Th1 activation via TLR2‐induced MyD88‐dependent pathway in vitro.

## 1 INTRODUCTION

Lung cancer causes an estimated 1.6 million deaths each year, being the leading cause of cancer‐related deaths in the world (Ostrowski, Marjański, & Rzyman, 2018). Although there are many drugs for lung cancer treatment on the market (Katayama, 2018; Lategahn, Keul, & Rauh, 2018), most of the tumor treatment drugs have strong toxic and side effects, which makes the patient's body function suffer from different degrees of damage (Camidge & Doebele, 2012; Chen et al., 2016). In recent years, immunotherapy of lung cancer has become a new research hot spot. It can enhance the immune function of the patient's body, exert an antitumor effect, and improve the tumor treatment effect (Reck, 2017; Wu, Kao, & Lai, 2018). The cells that mediate the body's antitumor immunity are mainly T cells and macrophages.

Previous studies have indicated that macrophages have the effect of killing tumor cells (Bayik, Tross, & Klinman, 2018; Zheng et al., 2018). Macrophages mainly differentiate into M1 and M2, and play different roles in tumor development (den Breems & Eftimie, 2016). M1 macrophages can secrete IL‐12, NO, ROI and IL‐1, IL‐6, TNF‐α, and other pro‐inflammatory cytokines, which can induce Th1‐type cell activation to a certain extent (Henao Agudelo et al., 2017; Rahat & Jivan, 2016; Zhang et al., 2016). T cell‐mediated cellular immunity is the main mechanism of the body's antitumor effect. T cells can be divided into two subgroups of CD4^+^ T cells and CD8^+^ T cells. Early studies have found that CD4^+^ T cells can be divided into two cell subpopulations, Th1 cells and Th2 cells, which play different roles in the development of tumors (Hirahara & Nakayama, 2016; Tsuda et al., 2017). After activation, Th1 cells mainly secrete cytokines such as IFN‐γ, IL‐12, and TNF‐α, which have significant anti‐intracellular bacteria, viral infections, and antiviral effects (Hwang, Kim, Lee, & Lee, 2012; Soleimani, Jameie, Barati, Mehdizadeh, & Kerdari, 2014).

The protein of silkworm pupae has been thought to be a new available source of high‐quality protein that contains all the amino acids needed by the human body (Zhou & Han, 2006). The silkworm peptide can be enzymatically hydrolyzed from silkworm protein. At present, the research on silkworm peptide mainly focuses on the physical properties and nutritional function analysis of silkworm peptide. There is little research on its immunity‐related function (Nakahara, Kanamori, Kiuchi, & Kamimura, 2003; Wang, Wang, Liu, & Jin, 2017). There is no relevant research on the mechanism of the medicinal value of silkworm peptide.

In this study, the immunity‐related silkworm peptide was studied in depth. Lewis lung cancer xenograft model mice were established, and different doses of silkworm peptides were used for in vivo immune. The molecular mechanism of activation TH1 and polarization of M1 macrophages were studied for the first time, which laid the experimental foundation for its therapeutic use or adjuvant drug use.

## 2 MATERIALS AND METHODS

### 2.1 Preparation of silkworm peptide

Silkworm protein was enzymatically hydrolyzed with alkaline protease (China Pangbo Biological Co., Ltd.) at 55°C for 2.5 hr with solid–liquid ratio of 1:7 and enzyme concentration of 7%. In this study, Sephadex G‐25 cation exchange chromatography column was used to separate and purify the silkworm peptide after debitterizing treatment. In this experiment, the molecular weight of the silkworm peptide after separation and purification was determined. Compared with the electrophoresis map of low molecular weight protein standards, the molecular weight of silkworm protein was greatly reduced after alkaline protease hydrolysis. The molecular weight of the active peptide after enzymatic hydrolysis was around 3 kDa.

### 2.2 Establishment of Lewis lung cancer mouse model and in vivo immunization

Twenty BALB/C mice (18–20 g) were randomly selected, and 0.2 ml (2 × 10^6^ cells) of LLC1 cells was injected into the right side of each mouse. Mice were immunized 3 days later. The specific method is as follows: 20 mice were classified into four groups (*n* = 5): PBS control group (400 μl PBS), low‐dose group (10 μg silkworm peptide), medium‐dose group (20 μg silkworm peptide), and high‐dose group (50 μg silkworm peptide).

PBS and silkworm peptide were subcutaneously injected at the back of the neck and bilateral inguinal injection. The second immunization was performed in the same manner at 7 days after the first immunization. Mice were received immunization for twice.

### 2.3 Mouse spleen lymphocyte proliferation detected by WST1 test

The Lewis lung cancer model mice were sacrificed by cervical dislocation. The spleen lymphocytes were isolated from the mouse lymphocyte separation solution, and 1 × 10^6^ cells per well were cultured in 96‐well plates for 48 hr. Each group had three duplicate wells. They were cultured in DMEM medium (containing 10% (v/v) fetal bovine serum, 100 U/ml penicillin, 100 μg/ml streptomycin); digested with trypsin, papain compound protease, and alkaline protease hydrolysate at a concentration of 20 μg/ml, respectively; and incubated for 48 hr at 37°C in a 5% CO_2_ incubator. One hundred microliter of the cell culture supernatant was aspirated, 10 μl of WST1 reagent was added, and A450 was measured at 37°C for 1 hr in the dark. Cell proliferation ability was expressed as relative cell viability: relative cell viability = (A450_experimental group_−A450_control group_)/A450_control group_.

### 2.4 Levels of IFN‐γ and IL‐4 detected by ELISA

The spleen lymphocytes of the Lewis lung cancer model mice were isolated and cultured for 48 hr, and the cell culture supernatant was collected. The levels of IFN‐γ and IL‐4 in the cell culture supernatant were measured according to the ELISA kit instructions.

### 2.5 Phagocytosis test of mouse peritoneal cavity macrophages

The mice were sacrificed by cervical dislocation. The 5 ml of PBS was injected into the abdominal cavity of the mouse using a syringe, and mice were gently massaged at the injection site for 2 min. The peritoneal fluid was aspirated using a pipette, centrifuged at 137 *g* for 5 min, and washed three times with 5 ml PBS at 137 *g* for 5 min. IMDM medium was added into 96‐well plates at 4 × 10^5^ cells per well. After incubated for 3 hr at 37°C in a 5% CO_2_ cell incubator, the unattached cells were removed with PBS. The cells were again cultured in a CO_2_ cell incubator for 48 hr. Incubation was continued for 1 hr by adding 100 μl of 0.075% neutral red (pH 7.4). It was washed three times with 200 μl/well PBS, and 100 μl of lysate was added into each well at 37°C for 2 hr. Microplate reader was measured at A540 nm. Levels of NO, IL‐6, IL‐1β, IL‐10, and IL‐12 in peritoneal macrophages of mice were measured by ELISA.

### 2.6 Isolation and purification and activation of CD4^+^ T cells

The cell concentration was adjusted to 25 × 10^6^ cells/ml using IMDM medium. The CD4^+^ T cells were isolated and purified according to the instructions of the CD4^+^ T‐cell immunomagnetic beads positive sorting kit. The specific method was as follows: 5 μl antibody was added per 1 × 10^6^ cells and incubated on ice for 30 min, and the tube was shaken every 5 min to allow the cells to fully mix with the antibody. The antibody not bound to the cells was completely removed washed with medium. An equal volume of immunomagnetic beads was added into the antibody, incubated on ice for 30 min, and shaken once every 5 min. The medium was added to calculate a cell concentration of 30 × 10^6^ cells/ml. One milliliter of the cell suspension was pipetted into the EP tube, and the EP tube was placed on the magnetic stand for 25 min. The supernatant was removed. The cells were mixed with 1 ml of medium, and the above steps were repeated twice. Flow cytometry was used to detect the purity of CD4^+^ T cells, and the cell purity shall be >95%.

And 100 μl of CD3 antibody (5 μg/ml) per well was coated in 96‐well plates at 4°C overnight. The purified CD4^+^ T cells were plated in the 96‐well plate at 1 × 10^6^ cells/well and incubated for 48 hr at 37°C in IMDM medium (containing 10% calf serum, 100 U/ml penicillin, 100 U/ml streptomycin). CD4^+^ T cells could be fully activated.

### 2.7 CD4^+^ T‐cell proliferation detected by WST1 test

The activated CD4^+^ T cells were collected and cultured in a 96‐well plate at 1 × 10^6^ cells/well. The different concentrations of silkworm peptides (5, 10, and 20 μg/ml) were added to stimulate cells in vitro, and three replicate wells were set in each group. They were incubated for 48 hr at 37°C in a 5% CO_2_ cell culture incubator. The effects of different concentrations of silkworm peptides on the proliferation of mouse CD4^+^ T cells were examined in vitro by WST1 test.

### 2.8 Detection of IFN‐γ and IL‐4 from mouse CD4^+^ T cells

Activated mouse CD4^+^ T cells were stimulated in vitro with 5, 10, and 20 μg/ml silkworm peptides, and cultured in 96‐well plates for 48 hr at a rate of 1 × 10^6^ cells per well. The cell culture supernatant was collected, and the concentrations of IFN‐γ and IL‐4 in the cell culture supernatant were measured by ELISA.

### 2.9 Detection of TLR2 mRNA expression in mouse CD4^+^ T cells by qRT‐PCR

The TLR2 mRNA primer sequence is as follows: Forward: 5′‐GCTTCGTTGTTCCCTGTGTT‐3′; Reverse: 5′‐AGTGGTTGTCGCCTGCTT‐3′. Activated CD4^+^ T cells were stimulated in vitro and cultured at 37°C for 2 hr. The 1.0 × 10^6^ cells were collected from each group and placed in an EP tube. And 0.5 ml Trizol was added, mixed well, and placed at room temperature for 5 min; 100 μl of chloroform was added, mixed vigorously for 15 s, placed at room temperature for 3 min, and centrifuged at 13,685 *g* for 10 min at 4°C. The upper aqueous phase was added into a new EP tube, and an equal volume of isopropanol was added, placed at room temperature for 10 min, and centrifuged at 13,685 *g* for 10 min at 4°C. The supernatant was removed; 5 ml of 75% ethanol (DEPC water configuration) was added, mixed, and centrifuged at 7,698 *g* for 5 min at 4°C. It was dried for 5 min, and 15 μl of DEPC water was added to dissolve the RNA.

A total of 1 μg of RNA and 1 μl of Oligod (T) were added to DEPC water to make a total volume of 10.5 μl at 70°C water bath for 10 min and quickly placed on ice for 3 min. The 4 μl M‐MLV buffer, 4 μl dNTP, 1 μl RTase M‐mlv, and 0.5 μl RTase inhibitor were added, and the following reactions were carried out in a PCR instrument: 42°C, 1 hr; 70°C, 15 min. After the reaction, it was cooled on ice for 20 min. The 1 μl of cDNA that was diluted 10 times, 0.6 μl of upstream and downstream primers, 10 μl of SyBR, and 7.8 μl of ddH_2_0 were added, and qRT‐PCR product was performed in a qRT‐PCR instrument.

### 2.10 Western blot analysis of TLR2 signaling pathway‐related protein expression

The 1.5 × 10^6^ activated CD4^+^ T cells were collected from each group, and 150 μl of cell lysate was added, lysed on ice for 30 min, and centrifuged at 13,685 *g* at 4°C. The supernatant was collected for 15 min. Protein concentration was determined according to the BCA kit instructions. 20 μg of protein was added into 5× loading buffer, boiled at 100°C for 5 min, and separated by SDS‐PAGE. The protein was transferred into the PVDF membrane for 2 hr and then placed in 5% skim milk powder (PBST dissolved) on a shaker at room temperature for 2 hr. After blocked by 5% skim milk powder, the corresponding primary antibodies were TLR2, TLR4, TLR9, MyD88, TRIF, TRAF3, TRAF6, IκB, p‐IκB, and β‐actin. β‐actin was used as an internal reference. It was shaken for 2 hr at room temperature (or overnight at 4°C) and washed three times with PBST. The 1:10,000 diluted goat anti‐mouse IgG‐HRP or goat anti‐rabbit IgG‐HRP was added, shaken at room temperature for 2 hr, and washed three times with PBST. ECL reagent was used to develop color, and images were acquired according to the ECL detector operating instructions.

### 2.11 Statistical analysis

Statistical analysis was performed on the relevant data using the SPSS 17.0 software processing system, and *p *<* *0.05 was defined to be a significant difference. The *t* test was used for comparison between the different groups, and the data were expressed by $\overset{¯}{x}$ ± *SD*. All data in this study were average results from three repeated tests.

## 3 RESULTS

### 3.1 Effect of silkworm peptide on the growth of Lewis lung cancer xenografts in mice

**Figure 1:**
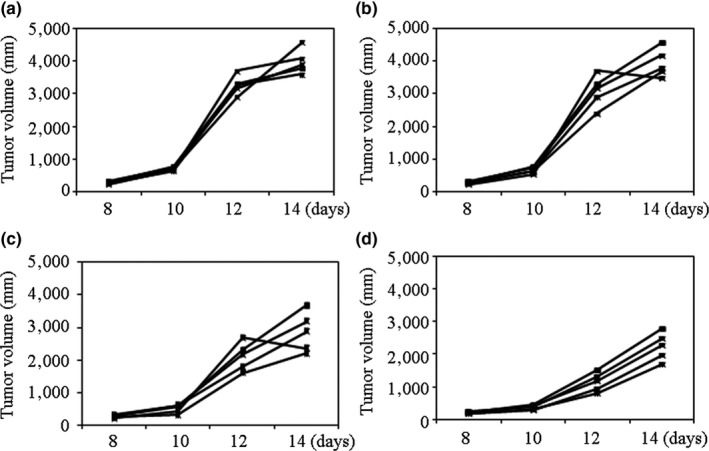
The inhibiting effect of different doses of silkworm peptides on tumor growth in Lewis lung carcinoma‐bearing mice among control (a) , low dose (b), medium dose (c), and high dose groups (d)

The volume of Lewis lung cancer xenografts in mice was compared (Figure 1). There was no significant change in tumor volume in the low‐dose group compared with the control group. The growth rate of Lewis lung cancer xenografts in mice of the medium‐dose group was significantly reduced, and the tumor volume was significantly smaller than that of the control group on the 14th day. The Lewis lung cancer xenografts in the high‐dose group had the smallest volume. The above results suggested that the silkworm peptide could significantly inhibit the growth of Lewis lung cancer xenografts, and the effect was in a concentration‐dependent manner.

### 3.2 Effect of silkworm peptide on spleen lymphocyte proliferation in mice

**Figure 2:**
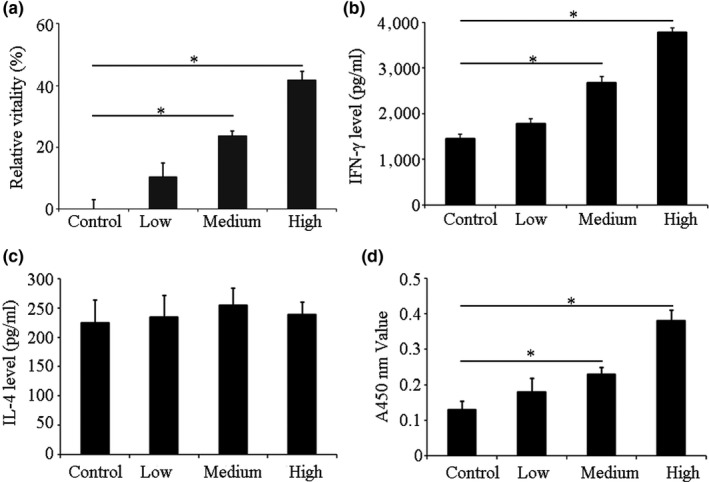
Effects of silkworm peptides on mice spleen lymphocytes and peritoneal macrophages. (a) Effect of silkworm peptides on the proliferation of Lewis lung carcinoma mice spleen lymphocytes detected by WST1. (b) Effect of silkworm peptides on IFN‐γ level of Lewis lung carcinoma‐bearing mice spleen lymphocytes detected by ELISA. (c) Effect of silkworm peptides on IL‐4 level of Lewis lung carcinoma‐bearing mice spleen lymphocytes detected by ELISA. (d) Effect of silkworm peptides on phagocytic activity of peritoneal macrophages in Lewis lung carcinoma‐bearing mice. * Indicated *p *<* *0.05 compared with control group

The relative vitality of spleen lymphocytes in the low‐dose immunized mice was 10.37%, and the difference was not statistically significant compared with the control group (*p *>* *0.05). The relative vitality value of spleen lymphocytes in the medium‐dose group was 23.61% and was significantly higher than the control group (*p *<* *0.05). The relative cell viability of the high‐dose group was the highest, which was 41.68%, suggesting that silkworm peptide could induce the proliferation of mouse spleen lymphocytes, and the effect was in a concentration‐dependent manner (Figure 2a).

### 3.3 The levels of IFN‐γ and IL‐4 in spleen lymphocytes of Lewis lung cancer model mice

The IFN‐γ level in the spleen lymphocyte supernatant of the Lewis lung cancer model mice immunized in the control group (784.54 pg/ml) was lower than the low‐dose group (832.67 pg/ml) (*p *>* *0.05). The IFN‐γ levels in the medium‐dose group (1,363.26 pg/ml) and high‐dose group (1,895.42 pg/ml) were significantly higher than the control group (*p *<* *0.05) (Figure 2b). The levels of IL‐4 were no significant change among different groups (Figure 2c). These results suggested that silkworm peptide could induce IFN‐γ secretion from lymphocytes of Lewis lung cancer model mice, and have no effect on the IL‐4 secretion.

### 3.4 Effect of silkworm peptide on the activity of peritoneal macrophages in Lewis lung cancer model mice

The experimental results were shown in Figure 2d. Compared with the control group, the A540 nm of the other three groups was increased to different extents, suggesting that silkworm peptide could increase the phagocytic activity of peritoneal macrophages in Lewis lung cancer model mice. However, there was no statistically significant difference in the phagocytic activity between the low‐dose group and the control group (*p *>* *0.05). Compared with the control group, the A540 nm in the medium‐dose group was significantly increased, suggesting that the phagocytic activity of the peritoneal macrophages in the medium‐dose group was significantly increased. The A540 nm in the high‐dose group was 0.38. The above experimental results suggested that silkworm peptide could significantly increase the phagocytic activity of peritoneal macrophages in Lewis lung cancer model mice, and the effect was in a concentration‐dependent manner.

### 3.5 Effect of silkworm peptide on the transformation of peritoneal macrophages into M1 type in Lewis lung cancer model mice

**Figure 3:**
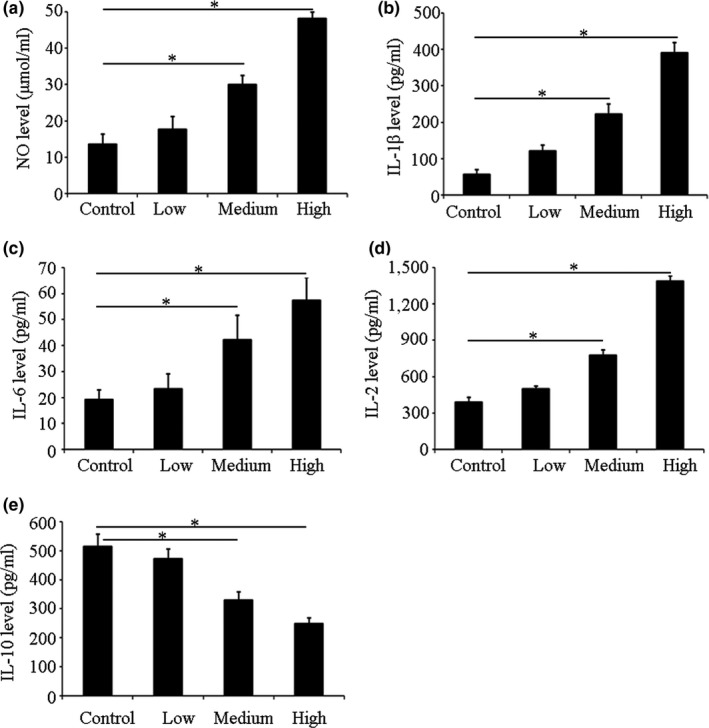
NO (a), IL‐6 (b), IL‐1β (c), IL‐12 (d), and IL‐10 (e) levels of Lewis lung carcinoma‐bearing mice macrophage after immunized with silkworm peptides. * Indicated *p *<* *0.05 compared with control group

Compared with the control group, different doses of silkworm peptide groups could increase the secretion of NO, IL‐6, IL‐12, and IL‐1β to varying degrees. In the low‐dose group, the levels of NO, IL‐6, IL‐12, and IL‐1β in mouse macrophage culture supernatant were not significantly increased compared with the control group, and the difference was not statistically significant (*p *>* *0.05). The levels of NO, IL‐6, IL‐12, and IL‐1β in the culture supernatant of the immunized mice in the medium‐dose group and high‐dose group were significantly higher than the control group (*p *<* *0.05) (Figure 3). Results indicated that the silkworm peptide could promote the secretion of M1 pro‐inflammatory cytokines by mouse macrophages, and the effect was in a concentration‐dependent manner. The level of IL‐10 in the supernatant of the control group (513.65 ± 29.37 pg/ml) was not significantly different from the low‐dose group (487.65 ± 15.13 pg/ml) (*p *>* *0.05), and was significantly lower than the medium‐dose group (304.97 ± 31.24 pg/ml) and high‐dose group (257.12 ± 23.62 pg/ml) (*p *<* *0.05) (Figure 3e). The above experimental results suggested that the silkworm peptide could effectively reduce the secretion of M2 type cytokines in mouse macrophages, and the effect was concentration dependent. Combining these results, it suggested that silkworm peptide could induce polarization of M1 macrophages in Lewis lung cancer model mice and inhibit polarization of M2 macrophages.

### 3.6 Effect of silkworm peptide on the proliferation of mouse spleen lymphocytes in vitro

The relative viability of the cells in 5 μg/ml silkworm protein group was 9.87% and was not significantly different from that of the control group. Compared with the control group, 10 and 20 μg/ml of silkworm peptides could significantly increase the relative viability (25.13% and 44.59%) of lymphocytes, and the relative viability value was gradually increased with the increase in active peptide concentration. The above results suggested that the silkworm peptide could induce the proliferation of mouse spleen lymphocytes, and the effect was in a concentration‐dependent manner.

### 3.7 Effect of silkworm peptide on CD4^+^ T‐cell subsets in mouse lymphocytes

**Figure 4:**
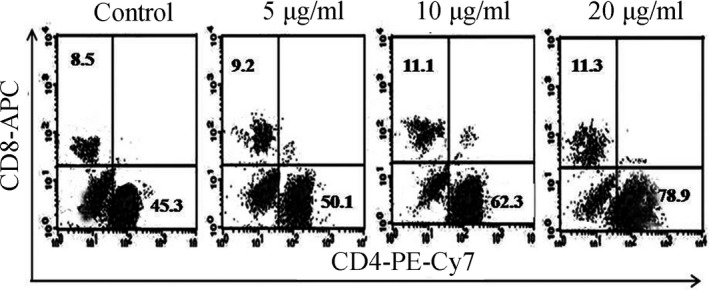
The flow cytometry of CD4^+^ T cell and CD8^+^ T cell stimulated with silkworm peptide

To further detect the cell subsets induced by silkworm peptides, mouse spleen lymphocytes were stimulated for 48 hr after different concentrations of silkworm peptide (5, 10, 20 μg/ml). Cells were stained with anti‐CD3‐PE, anti‐CD4‐PE‐cy7, and anti‐CD8‐APC antibodies. The percentage of CD4^+^ T cells and CD8^+^ T cells in this range were detected by flow cytometry with CD3^+^ T cells (Figure 4). In the control group, the proportion of CD3+ T cells in the CD8+ T cells was 8.5%. After stimulation with the silkworm peptide, the proportion of CD8^+^ T cells (5 μg/ml group, 9.2%; 10 μg/ml group, 11.1%; 20 μg/ml group, 11.3%) were not significantly different compared with the control group. The proportion of CD4^+^ T cells in the control group was 45.3%. After stimulation with 5 μg/ml silkworm peptide, the proportion of CD4^+^ T cells was increased to 50.1%, which was not statistically significant (*p *>* *0.05). After stimulation with 10 μg/ml silkworm peptide, the proportion of CD4^+^ T cells was increased to 62.3%, which was significantly different from the control group (*p *<* *0.05). The proportion of CD4^+^ T cells in the 20 μg/ml group was the highest (78.9%), which was significantly higher than the control group and the other two experimental groups. These results suggested that the silkworm peptide could significantly induce the proliferation of CD4^+^ T cells in mouse lymphocyte.

### 3.8 Effect of silkworm peptide on IFN‐γ secretion from mouse spleen lymphocytes in vitro

**Figure 5:**
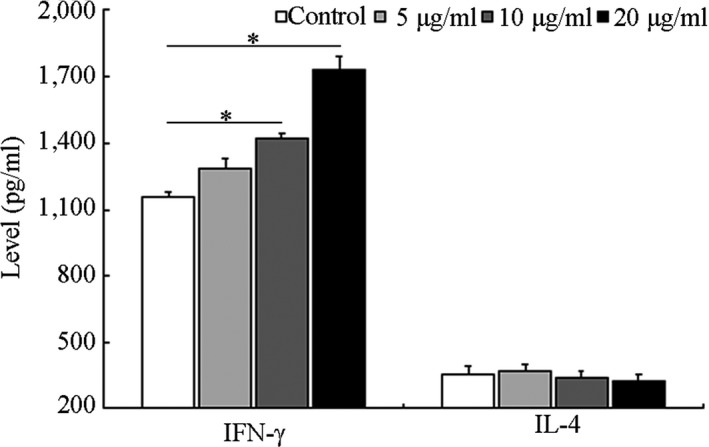
The IFN‐γ and IL‐4 levels of spleen lymphocytes stimulated with silkworm peptide**s** analyzed by ELISA**.** * Indicated *p *<* *0.05 compared with control group

Compared with the control group, 10 μg/ml silkworm peptide could significantly promote the secretion of IFN‐γ from mouse spleen lymphocytes, and the effect was obvious with the increase in silkworm peptide concentration. There was no significant change in the secretion level of IL‐4 in the supernatant of the control group, 5, 10, and 20 μg/ml groups (Figure 5). The above experimental results suggested that high concentration of silkworm peptide could significantly promote the secretion of IFN‐γ from mouse spleen lymphocytes.

### 3.9 Proliferation of mouse CD4^+^ T cells

**Figure 6:**
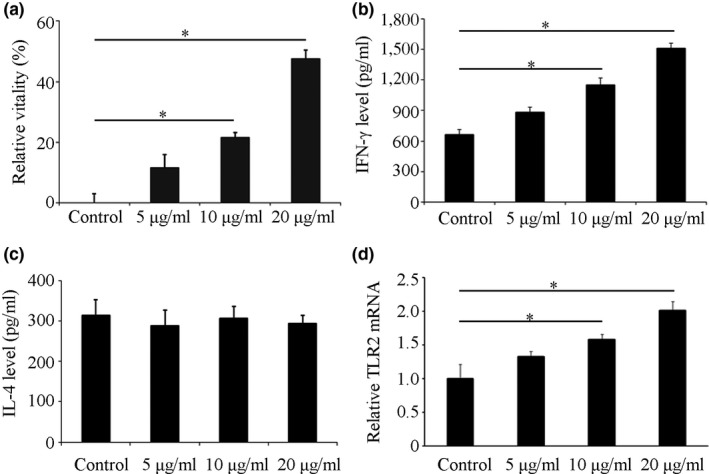
Effects of silkworm peptides on CD4^+^ T cells. (a) Effect of silkworm peptides on the proliferation of activated CD4^+^ T cells detected by WST1. (b) Effect of silkworm peptides on IFN‐γ level of activated CD4^+^ T cells detected by ELISA. (c) Effect of silkworm peptides on IL‐4 level of activated CD4^+^ T cells detected by ELISA. (d) Effect of silkworm peptides on TLR2 mRNA expression of activated CD4^+^ T cells detected by qRT‐PCR.* Indicated *p *<* *0.05 compared with control group

The immunomagnetic beads were used to sort the mouse spleen CD4^+^ T cells, the cells were labeled with CD4‐PE‐cy7 antibody, and the purity of CD4^+^ T cells was tested by flow cytometry. The percentage of CD4^+^ T cells was 97.1%, which met the experimental requirements. After stimulation with 5 μg/ml silkworm peptide, the relative viability was 11.45%. Compared with the control group, the difference was not statistically significant (*p *>* *0.05). Compared with the control group, the relative viability values in 10 μg/ml (21.53%) and 20 μg/ml groups (47.63%) were statistically significant (*p *<* *0.05) (Figure 6a). The above experimental results suggested that the silkworm peptide could directly induce the proliferation of activated mouse CD4^+^ T cells, and the effect was in a concentration‐dependent manner.

### 3.10 Effect of silkworm peptide on Th1 activation in mouse CD4^+^ T cells

The concentration of IFN‐γ in 5 μg/ml group (886.31 pg/ml) was not statistically significant difference from the control group (*p *>* *0.05). The concentrations of IFN‐γ in 10 and 20 μg/ml groups were increased significantly and were statistically significant from control group (*p *<* *0.05) (Figure 6b). In addition, IL‐4 concentrations were no significant differences among all groups (Figure 6c), indicating that the silkworm peptide had no effect on the secretion of IL‐4 by CD4^+^ T cells. These results suggested that the silkworm peptide could directly induce Th1 activation in mouse CD4^+^ T cells, and the effect was in a concentration‐dependent manner.

### 3.11 Activation of TLR2‐mediated MyD88‐dependent pathway

Compared with the control group, the expression level of TLR2 mRNA was increased in different degrees after activation of CD4^+^ T cells stimulated with silkworm peptide. Compared with the control group, the increase of TLR2 mRNA in the 5 μg/ml group was not significant (*p *>* *0.05); the TLR2 mRNA in the 10 μg/ml group and 20 μg/ml groups were statistically significantly higher than the control group (*p *<* *0.05) (Figure 6d).

**Figure 7:**
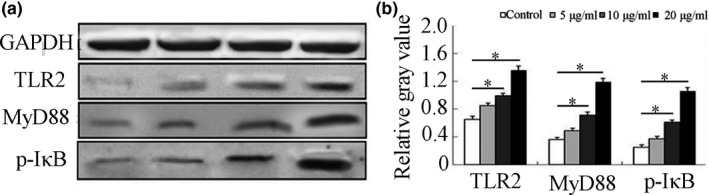
Effect of silkworm peptides on TLR2, MyD88, and IκB expressions of activated CD4^+^ T cells detected by Western blot

Western blot analysis results were shown in Figure 7. The expression level of TLR2 protein was increased after stimulation with silkworm peptide. The expression level of TLR2 protein in 20 μg/ml group was the highest, which was consistent with the results of qRT‐PCR. In addition, Western blot was used to detect the protein expression levels of MyD88 and p‐IκB. Compared with the control group, the protein expression levels of MyD88 and p‐IκB in 10 μg/ml group and 20 μg/ml groups were increased to a certain extent and were significantly increased compared with the control group (*p *<* *0.05). The above experimental results suggested that the silkworm peptide could directly induce Th1 activation in vitro by activating the TLR2‐mediated MyD88‐dependent pathway.

## 4 DISCUSSION

The antitumor effect of silkworm peptide and its mechanism of action were explored in this study. When the Lewis lung cancer model mice were immunized with 20 μg/mouse of silkworm peptide, a better tumor growth inhibition effect was obtained, and the effect was in a concentration‐dependent manner. Studies have shown that bioactive peptides from a variety of sources can effectively inhibit tumor cell proliferation (Li et al., 2013). This study also found that silkworm peptide could effectively inhibit the growth of mouse Lewis lung cancer xenografts. However, there are currently few studies on the immunomodulatory function of the silkworm peptide. A large number of studies have shown that Th1 cells play a key role in the development of tumors and are ideal subpopulations of tumor immunotherapy (Fernandez et al., 2007). In this study, silkworm peptide could induce Th1 activation and M1 type, which was dose‐dependent. The silkworm peptide could play role in inhibiting tumor growth, and it may be related to TLR2‐induced MyD88‐dependent pathway in vitro.

Macrophages are important constituent cells of innate immunity and play a wide‐ranging role in the body's fight against foreign antigens (Bryan et al., 2018; Curtale, 2018). In recent years, studies have found that macrophages play an important role in the development and progression of malignant tumors in addition to the traditional immunomodulatory effects (Furudate et al., 2017; Nie et al., 2017; Tanita et al., 2017). Macrophages can differentiate into different activation types in different local microenvironments: classically activated macrophages (M1) and alternatively activated macrophages (M2). Different types of macrophages play different roles in the development of tumors. Studies have shown that different polarization types of mouse peritoneal macrophages secrete different kinds of cytokines during the growth process (Chi, Jiang, Lei, Shen, & Yang, 2016; Oishi et al., 2016). M1 macrophages mainly secrete NO, IL‐6, IL‐12 and TNF‐α, and other inflammatory factors (Wang et al., 2015). M2 macrophages secrete a higher concentration of the anti‐inflammatory factor IL‐10 (Li et al., 2017). Therefore, this study used ELISA to detect the NO, IL‐6, IL‐12, IL‐10, and IL‐1β levels in the culture supernatant of mouse peritoneal macrophages after treated with different doses of silkworm peptides in Lewis lung cancer model mice, and it was used to confirm the effect of silkworm peptide on the polarization type of mouse macrophages. Results indicated that silkworm peptide could induce polarization of M1 macrophages in Lewis lung cancer model mice and inhibit polarization of M2 macrophages.

Th1 activation of CD4^+^ T cells can be through two completely different pathways: One pathway is to induce Th1 activation through indirect action; that is, the antigen first activates antigen‐presenting cells in vivo, and then the activated antigen‐presenting cells extract the processed antigen to CD4^+^ T cells and induce Th1 activation; the other pathway is to induce Th1 activation by direct action; that is, the antigen does not need to interact with antigen‐presenting cells to directly induce Th1 activation in CD4^+^ T cells. In in vitro experiments, although the action pathway does not require the involvement of antigen‐presenting cells, it is necessary to first activate the CD4^+^ T cells using CD3 antibodies (Weiss, Raupach, Takeda, Akira, & Zychlinsky, 2004). Through the in vivo experiments in this study, we were unable to determine whether the silkworm peptide induced Th1 activation through direct or indirect effects. The previous study found that E. coli maltose‐binding protein can induce Th1 activation by activated CD4^+^ T cells through direct pathway (Fang et al., 2015). However, there is no relevant research on the effect of silkworm peptide on CD4^+^ T cells. It was hypothesized that silkworm peptides, also as protein components, may induce Th1 cell activation by acting on antigen‐presenting cells, and it was also possible to induce Th1 activation by direct action on CD4^+^ T cells. In the next experiment, silkworm pupa peptide was used to stimulate mouse spleen lymphocytes and purified CD4^+^ T cells in vitro, and the action of silkworm peptide on CD4^+^ T cells was further explored.

To determine whether silkworm peptide can induce Th1 activation, different concentrations of silkworm peptide (5, 10, 20 μg/ml) were first used to stimulate mouse spleen lymphocytes. The results of WST1 test showed that silkworm peptide could effectively induce lymphocyte proliferation; flow cytometry results found that silkworm peptide could increase the proportion of CD4^+^ T‐cell subsets, suggesting that silkworm peptide could promote the proliferation of CD4^+^ T cells. In this study, CD4^+^ T cells with higher purity were obtained by immunomagnetic beads sorting, and activated by CD3 antibody, and then, different concentrations of silkworm peptide stimulated activated CD4^+^ T cells. The above experimental results suggested that the silkworm peptide can directly induce Th1 activation in activated CD4^+^ T cells, and the effect was in a concentration‐dependent manner.

It has been reported that in addition to high expression on antigen‐presenting cells (APC), TLR2 is functionally expressed on CD4^+^ T cells and directly regulates the immune function of CD4^+^ T cells (Martino, Casetti, & Poccia, 2007). Current studies have shown that activation of the TLR2 signaling pathway directly induces Th1 activation in CD4^+^ T cells (Liu, Zhai, et al., 2017; Liu, Zhang, et al., 2017). The study found that in vitro stimulation of mouse spleen CD4^+^ T cells after activation with CD3 antibody using TLR2 agonist‐Pam3CKS for 48 hr, the concentration of IFN‐γ in cell culture supernatants could be significantly increased (Zhang, Sun, Wei, & Tian, 2005). Another study indicated that Pam3CKS stimulated mouse CD4^+^ T cells after CD3 and CD28 antibody co‐activation in vitro, and it could significantly increase IFN‐γ secretion and reduce IL‐4 secretion (Orr et al., 2013).

It was speculated that the silkworm peptide may also induce Th1 activation through the TLR2 signaling pathway. Therefore, Western blot and qRT‐PCR experimental techniques were used to detect changes in TLR2 mRNA and protein expression levels after silkworm peptide stimulation of CD4^+^ T cells. The results showed that different concentrations of silkworm peptide could significantly increase TLR2 mRNA and protein expression levels in vitro, and the effect was in a concentration‐dependent manner. It was suggested that silkworm peptide could induce Th1 activation through TLR2 signaling pathway.

TLR2 is expressed on the cell surface and must bind to TLR1 or TLR6 to form a dimer, and binds to the corresponding ligand to induce activation of the signaling pathway (Choteau et al., 2017). The downstream signal molecule of TLR2 is MyD88. After binding to the corresponding ligand, TLR2 first activates MyD88 molecule, which in turn induces the activation of downstream signaling molecules and exerts corresponding biological functions, and this activation pathway becomes MyD88‐dependent pathway (Liu, Zhai, et al., 2017; Liu, Zhang, et al., 2017). The study has shown that if the TLR2 gene or MyD88 gene was knocked out, the mice were stimulated with the TLR2 agonist, and they were unable to induce DC cell maturation or induce B‐cell activation (Fukata et al., 2008). Western blot results showed that the expression level of MyD88 protein in TLR2 agonist‐stimulated group was significantly increased in this experiment, suggesting that TLR2 could directly induce Th1 activation in CD4^+^ T cells by activating MyD88‐dependent pathway in vitro. This was highly consistent with the experimental results obtained in this study. In this study, results showed that stimulation of activated CD4^+^ T cells with different concentrations of silkworm peptide not only significantly up‐regulated the expression of TLR2 protein, but also up‐regulated the activation of MyD88 and I‐kB. It was suggested that the TLR2‐mediated MyD88‐dependent pathway played an important role in the Th1 activation induced by the silkworm peptide. However, the specific reasons for the induction of Th1 activation by MyD88‐dependent pathway are still unclear. Some scholars believe that this effect of MyD88 molecule may be related to its ability to induce the activation of IL‐1β and IL‐18 receptor signaling pathway.

In conclusion, silkworm peptide could induce Th1 activation and M1 type polarization, which was dose‐dependent and was relative to the effect of silkworm peptide on inhibiting tumor growth. Silkworm peptide could directly induce M1 type polarization and Th1 activation via TLR2‐induced MyD88‐dependent pathway in vitro. However, this study was only performed in animal level, in order to verify the immune activity and molecular mechanism of silkworm peptide, and the further study is still needed.

## CONFLICT OF INTEREST

None.

## STATEMENT FOR GUIDELINES FOR HUMAN SUBJECTS

Not applicable.

## ETHICS APPROVAL

This study is approved by the Ethics Committee of Jiangsu University of Science and Technology.
